# Supplementary material for: Type 2C Protein Phosphatase MoPtc6 Plays Critical Roles in the Development and Virulence of Magnaporthe oryzae
Source: J Fungi (Basel). 2025 Apr 24;11(5):335. doi: 10.3390/jof11050335 (PMC12113234; doi:10.3390/jof11050335)
Supplement: Supplementary file 1 [file jof-11-00335-s001.zip › jof-3565571-supplementary.pdf]

Supplementary Figures and Tables

***Journal name* :** Journal of fungi

MoPtc6, a type 2C protein phosphatase plays critical roles in the development and virulence of *Magnaporthe oryzae*

Frankline Otieno Jagero [1], Abah Felix [1], Yakubu Saddeeq Abubakar [1], Meilian Chen [2], Wilfred M. Anjago [3],
Hatungimana Mediatrice [3], Nkurikiyimfura Oswald [3], Osakina Aron [1], Wei Tang [1,3], Zonghua Wang [1,2,3,*] and Jules Biregeya [1,3,*]

1 Fujian Universities Key Laboratory for Plant-Microbe Interaction, College of Life Science, Fujian Agriculture and Forestry University, Fuzhou 350002, China; frankja23@gmai.com (F.O.J.); fabah11@gmail.com (A.F.); ay.saddeeq@yahoo.com (Y.S.A.); osakina.aron@yahoo.com (O.A.); tangw@fafu.edu.cn (W.T.)
2 Fuzhou Institute of Oceanography, Minjiang University, Fuzhou 350108, China;
meilian2019@mju.edu.cn (M.C.); mabechewilfred@gmail.com (W.M.A.); mediatunga@gmail.com (H.M.); nk.oswaldo@gmail.com (N.O.)
3 College of Plant Protection, Fujian and Agriculture and Forestry University, Fuzhou 350002, China
* Correspondence: wangzh@fafu.edu.cn (Z.W.); biregeyakayihura2020@gmail.com (J.B.)

1. **Figure s1** , **Southern blot assay**

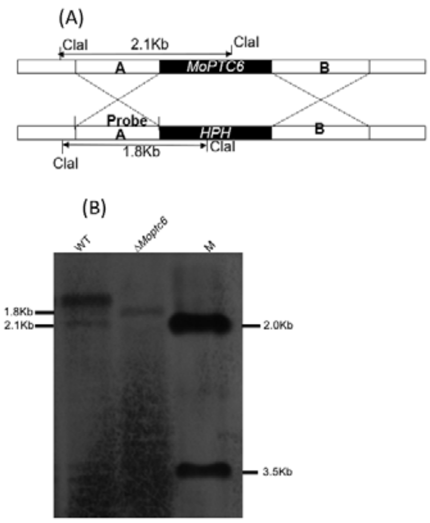

Figure S1, Represent Southern blotting assay. A-B. Δ*Moptc*6 display the replacement of opening reading frame regions (ORF) by insertion of hygromycin phosphotransferase gene (Hph).

Figure S2, Heatmap of the expressed genes in Δ*Moptc6* mutant and Wild type (Guy11)

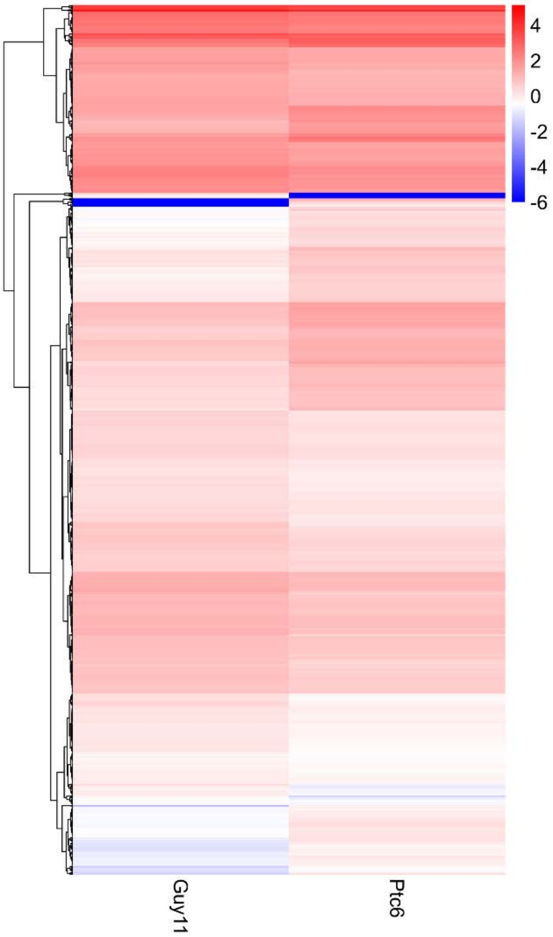

Table S1 fungal strains (wild-type and mutant) employed during this study

Strain	Genotype description	Reference
Guy11	Wild-type	This study
Δ*Moptc6*	*MoPTC6* deleted mutant from Guy11	This study

Table S2 List of primers used in this study

Primers	Sequence（5'-3'）	Application
MGG_MoPtc6 UA	GGGAGACACCACTGCGTAA	Verification of replacement of ORF with hygromycin

H853	GACAGACGTCGCGGTGAGTT	
MGG_ MoPtc6 AF	AAATCTCGGGTCAGTCTA	Amplification of A flagment
MGG_ MoPtc6 AR	TTGACCTCCACTAGCTCCAGCCAAGCCAATTGCGTGTTTGTTGAG	
MGG_MoPtc6 BF	GAATAGAGTAGATGCCGACCGCGGGTTGATACTTCAACTCTGGCTTAA	Amplification of B flagment
MGG_MoPtc6 BR	CTCTGGTCTCAGGTCAAG	
MGG_ MoPtc6 OF	CCACTTCCACAACTACTT	Verification of opening reading flame (ORF)
MGG_ MoPtc6 OR	GCTCTTGGTGATACTGAA	
	'	
HG-F	GAATAGAGTAGATGCCGACCGCGGGTT	Amplification HG flagment
HG-R	TTGACCTCCACTAGCTCCAGCCAAGCC	
MoPtc6.comF	agggaacaaaagctgggtaccTTGGCTAATTCTGTAAGC	Primers for complementation assay
MoPtc6.comR	gcccttgctcaccataagctt CCTTCTCCTCCCTCTTGGGT	
MGG_MoCON6 QF	AGCAGCACTCGAAGAAGGT	Primers for conidiation related genes
MGG_MoCON6 QR	ACATCGCCGCCATCAAAC	
MGG_MoCON1QF	AACCAATGCTTCCGATAT	
MGG_MoCON1QR	TGAGTCGTTGTAGTATGC	Primers for conidiation related genes
MGG_MoCON7 QF	CGATACGAAGAAATTGAG	
MGG_MoCON7 QR	GGATCTCCTTAAACTCTT	Primers for conidiation related genes

MGG_MoCON8 QF	ACCTCTTCCTCTTCTTCG	
MGG_MoCON8 QR	TTGGTCTGTTCGAGTGAT	
MGG_MoflbAQF	ACAACCTTACAACGATAC	
MGG_MoflbAQR	TAACATGCTAACAATTAACG	
MGG_MoflbC QF:	AAATGTTATGACGAAAGAG	
MGG_MoflbC QR:	TTGTGTTGAAAGTTGAAG	
